# Effect of Silica Nanoparticle Treatment on Adhesion between Tissue-like Substrates and In Vivo Skin Wound Sealing

**DOI:** 10.3390/jfb15090259

**Published:** 2024-09-09

**Authors:** Yeji Jeon, Tae Ryeol Kim, Eun Seo Park, Jae Hyun Park, Han Sung Youn, Dae Youn Hwang, Sungbaek Seo

**Affiliations:** 1 Department of Biomaterials Science (BK21 FOUR Program), College of Natural Resources and Life Science/Life and Industry Convergence Research Institute, Pusan National University, Miryang 50463, Republic of Korea; yeji000307@gmail.com (Y.J.); xofuf0701@naver.com (T.R.K.); geg9393@naver.com (E.S.P.); 2 Young Chemical Co., Ltd., 80-93, Golden root-ro, Juchon-myeon, Gimhae 50969, Republic of Korea; jhpark@youngchemical.co.kr (J.H.P.); hsyoun@youngchemical.co.kr (H.S.Y.)

**Keywords:** inflammation, silica nanoparticles, surgical glue, tissue adhesion, wound healing

## Abstract

Silica nanoparticles are innovative solutions of surgical glue that can readily adhere to various tissue-like substrates without the need for time-consuming chemical reactions or ultraviolet irradiation. Herein, 10 nm-sized silica nanoparticle (SiNP_10_) treatment exhibited maximum adhesion strength in the porcine heart tissue model, which was approximately 7.15 times higher than that of the control group of non-treatment. We assessed the effects of silica nanoparticle treatment on in vivo skin wounds by scoring tissue adhesion and inflammation using histological images. Compared to the commercial cyanoacrylate skin adhesive (Dermabond), suppression of inflammatory cytokine levels in the incision wound skin was observed. We further quantified the expression of angiogenic growth factors and connective tissue formation-related proteins. On day 5 after wound closing treatment, the expression levels of PDGF-BB growth factor were significantly higher in SiNP_10_ treatment (0.64 ± 0.03) compared to Dermabond (0.07 ± 0.05). This stimulated angiogenesis and connective tissue formation in the skin of the incision wound may be associated with the promoting effects of SiNP_10_ treatment on wound closure and tissue adhesion.

## 1. Introduction

Traumatic injury is a leading cause of death in Europe and the United States [1]. Millions of surgical procedures are performed annually for injury treatment [2]. Surgical procedures require rapid wound closure techniques to reduce inflammation, infection, and scarring. Accordingly, the global wound care market was approximately $12 billion in 2020 and is estimated to grow to $18.7 billion by 2027 [3].

Sutures and staples are standard biomaterials used for wound closure [4,5,6]. Sutures are widely used for wound treatment; however, they are difficult to apply in minimally invasive surgeies and pose a high risk of infection [7]. Additionally, the tension applied to the wound after suturing can cause complications such as inflammation and scar enlargement [6,7,8]. Staples are simpler than sutures and allow quick closure; however, the risk of infection and wound complications are higher with staples than with sutures [5,9].

Polymer-based adhesives have been suggested because of their good contact with surfaces and the ability to maintain the fracture of the wound area by dissipating energy under pressure. However, they require complex processes, such as in situ polymerization [10,11], and the active oxygen generated during polymerization can damage healthy tissues [12]. Polymer-based adhesives exhibit moisture-absorbing properties, a high expansion rate, and can suppress nerves, which limits their usage [12,13]. Accordingly, adhesive materials with convenience, low toxicity, and high wound-sealing ability are required.

Biological adhesives have been clinically introduced as wound-closing materials. The adhesive is manufactured from cyanoacrylate, fibrin, and chitosan [6]. An adhesive is a fluid or semifluid mixture that can easily seal irregular wounds because it acts as an interface by bonding the edge surfaces of the wound [14]. However, these adhesives exhibit a low degree of degradation, remain in the tissue even after wound healing, exhibit high cytotoxicity, and cause allergic reactions in some patients [12,15,16]. In addition, in environments with large amounts of exudate, the adhesive strength may be low [14]; hence, their application is limited.

Recently, silica nanoparticles (SiNP) and silver nanoparticles have been used as tissue adhesives and glue [17,18,19]. Studies have been conducted on the adhesion between hydrogels and tissues following SiNP treatment. The biomacromolar network chains in the tissue can be adsorbed on the surface of the SiNP, making it possible for them to act as a bridging connector between the hydrogel and tissue without chemical reactions [17,20]. In particular, SiNPs that are similar in size to the network mesh of hydrogels and tissues exhibit high adhesion [17] because multiple network chains can be adsorbed. The SiNPs can be used in biomedical applications with low toxicity and high biocompatibility and can be used as tissue adhesives [21,22].

In this study, smooth, spherical SiNPs were used to investigate their adhesive abilities according to their size and concentration. We measured the adhesive properties of the two types of substrates (polymer hydrogel, porcine liver, and heart) using SiNP treatment via lap shear tests. We further evaluated the wound closure and healing capability of SiNP treatment in vivo in rat skin by comparing the expression levels of inflammatory cytokines and tissue regeneration-contributing proteins between the suture- and commercial-skin adhesive-treated groups.

## 2. Materials and Methods

### 2.1. Materials

N,N-Dimethylacrylamide (DMA, 99%) was purchased from Tokyo Chemical Industry Co., Ltd. (Tokyo, Japan). *N*,*N*′-Methylenebisacrylamide (MBA, 99%), potassium persulfate (KPS, 99%), *N*,*N*,*N*′,*N*′-tetramethylethylenediamine (TEMED, 99%), Ludox^®^TM-30 (SiNP_10_, *9.6 ± 1.5 nm in diameter; 30 wt% aqueous suspension), Ludox^®^TM-50 (SiNP_30_, *29.3 ± 3.8 nm in diameter; 50 wt% aqueous suspension), gelatin (gelatin from porcine skin, gel strength 300, Type A) were purchased from Sigma Aldrich (St. Louis, MO, USA). Phosphate buffered saline (PBS) was purchased from Welgene (Gyeongsan, Republic of Korea). Porcine liver and heart tissues were purchased from BIOZOA (Seoul, Republic of Korea). *The size of SiNP was observed and defined by scanning electron microscopic (SEM) images.

### 2.2. Preparation of Poly (Dimethylacrylamide) (PDMA) and Gelatin Hydrogel

**PDMA hydrogel**. The DMA (1.485 mL), MBA (0.002 g), and KPS (0.041 g) were dissolved in 7.6 mL of de-ionized water (DI water) at room temperature (25 ± 2 °C) under nitrogen and stirred vigorously for 30 min. The TEMED (22.5 μL) was quickly added to the solution and stirred for 1 min. This solution (9 mL) was poured into a petri dish (SPL Petri Dishes 10060, SPL Life Sciences, Pochenon, Republic of Korea; 60 mm diameter × 15 mm height) and left at room temperature for 24 h.

**Gelatin hydrogel**. Gelatin powder was added to PBS (15% *w*/*v*) and mixed at 65 °C until dissolution. Liquid gelatin (9 mL) was poured into a petri dish and cooled at 4 °C for 24 h.

### 2.3. Lap Shear Adhesion Tests of SiNP Treated Hydrogels and Tissue Model

**Figure 1:**
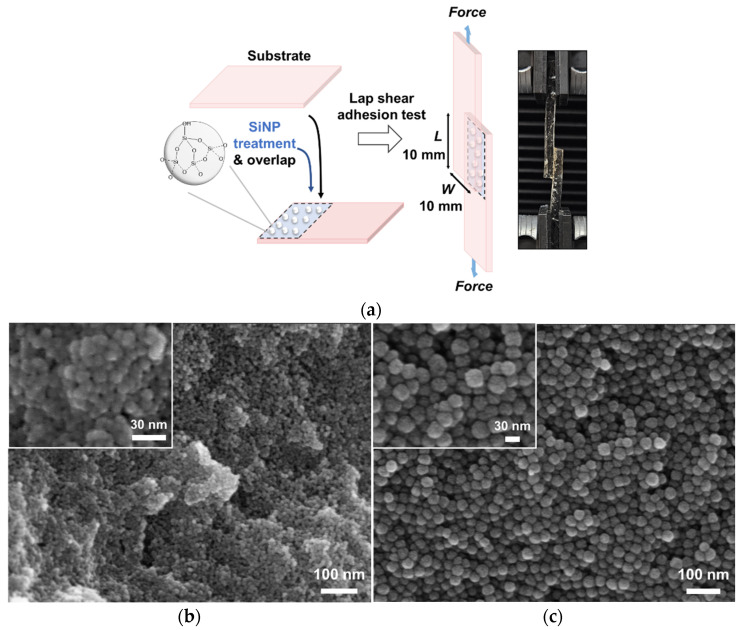

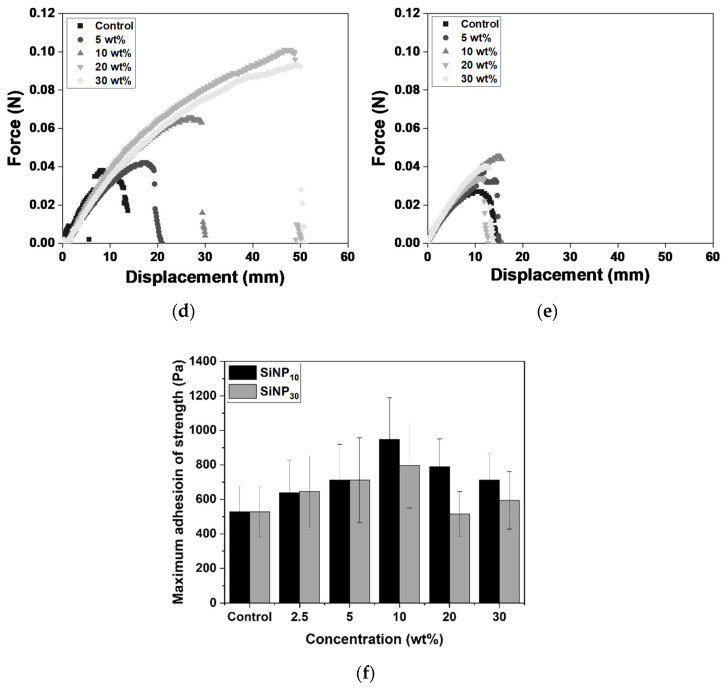
Adhesion tests of polymer hydrogels by treatment of silica nanoparticles (SiNP) at their interface. (**a**) Schematic illustration of lap shear adhesion test using SiNP treatment between substrates. Photograph of the adhesion test with poly (dimethylacrylamide) (PDMA) hydrogel substrates. The SEM image of the used (**b**) SiNP_10_ and (**c**) SiNP_30_; SiNP_10_, and SiNP_30_ means 10 nm and 30 nm in diameter-sized silica nanoparticles, respectively. Representative displacement-force curves were obtained from the adhesion tests according to the concentration of (**d**) SiNP_10_ and (**e**) SiNP_30_ treatment between two PDMA hydrogels. (**f**) Maximum adhesion strength depending on the size of SiNP treatment between two PDMA hydrogels. The data (*n* = 3) were presented as mean ± SD.

The tests were conducted using the PDMA hydrogel, gelatin hydrogel, porcine liver, and porcine heart (Figure 1a). The substrate was cut to dimensions of 35 × 10 × 5 mm (length × width × thickness). The SiNP_10_ and SiNP_30_ (20 μL) were applied to one side of the substrate and another substrate was adhered for 30 min. The adhesion strength profiles of the SiNP-treated substrates were measured using a Universal Testing Machine (UTM, A&D 5OOOH, Daegu, Republic of Korea) equipped with a 5 N load cell at a speed of 150 mm/min. The SiNP-treated area (overlapped between substrates) was 10 mm long × 10 mm wide with a dropping volume (0.20 μL/mm^2^).

### 2.4. Animal Experiments

Sprague Dawley (SD) rats were handled at the Pusan National University Laboratory Animal Resources Center, accredited by the Korea Food and Drug Administration (KFDA) (Accredited Unit Number-000231) and AAALAC International, in accordance with the National Institutes of Health guidelines (Accredited Unit Number-0001525). The animal experimental protocol used in this study was approved by the Institutional Animal Care and Use Committee (IACUC) of Pusan National University (PNU-2023-0277). Male SD rats (7-weeks old) were provided by Samtako BioKorea Inc. (Osan, Republic of Korea). They were housed under specified pathogen-free (SPF) conditions and a strict light cycle (lights on at 08:00 h and off at 20:00 h) at a temperature of 23 ± 2 °C, and relative humidity of 50 ± 10%. The rats had *ad libitum* access to a standard irradiated chow diet (Samtako BioKorea Inc., Osan, Republic of Korea) and water.

Briefly, SD rats (*n* = 30) were randomly divided into one of the following five groups: Control (*n* = 6), Suture (*n* = 6), Dermabond (*n* = 6), SiNP_10_ (*n* = 6), and SiNP_30_ (*n* = 6). The details of these groups are described below. All rats were anesthetized via intraperitoneal injection with an anesthetic mixture (4:1 ratio) containing 40 mg/kg Alfaxan (Jurox Pty Limited, Maitland, Australia) and 10 mg/kg Rompun (Bayer–Korea, Ltd., Seoul, Republic of Korea); subsequently, their dorsal hair was shaved using an animal hair clipper (hair clipper, Bodeum company, Osan, Republic of Korea). Full-thickness longitudinal incisions of 1.5 cm length and 3 mm depth were made on the dorsal skin using surgical disposable scalpels. After that, the incision wound was not treated in the Control group, whereas the incision wound was sutured in the Suture group (non-absorbable silk suture 2-0, AILEE Co., Ltd., Pusan, Republic of Korea). The Dermabond group was glued together in the regions of the incision wound with a commercial skin adhesive (DermabondTM mini, ETHICON, Inc., Raritan, NJ, USA). In the SiNP_10_ and SiNP_30_ groups, these incision wounds were applied with 100 μL of 10 nm sized SiNP and 30 nm sized SiNP solution using pipettes, respectively. Then, the treated sites were sealed with a transparent film (Tegaderm™ Transparent Film Dressing 1622W, 3M™, Los Angeles, CA, USA). The sealing with the film was conducted with all experimental groups to make the wound treatment to the sites thorough and reduce loss of treated materials. On the third and fifth days after wound adhesion, all rats were euthanized by trained researchers using an appropriate chamber with a gas regulator and CO_2_ gas with a minimum purity of 99.0%, according to the American Veterinary Medical Association Guidelines for the Euthanasia of Animals. Images of the incision wound regions were captured using a digital camera (SM-S908N, Samsung, Suwon, Republic of Korea), and wounded skin tissues were collected from the SD rats for further analyses.

### 2.5. Histological Analysis

Wound skin tissues collected from SD rats of subset groups were fixed in 10% formalin for 48 h, embedded in paraffin wax, and then sectioned into 4 μm thick slices. Skin sections were stained with a hematoxylin and eosin (H&E; Sigma-Aldrich, St. Louis, MO, USA) solution. Inflammation and adhesion in the wounded skin tissue were observed using an optical microscope (Leica Microsystems, Wetzlar, Germany). The scoring criteria for histopathological changes in wounded skin were applied by modifying the method used in a previous study [23]. Also, the wound distance was measured at 3–5 different sites under the scab using Image J 1.52a (NIH, Bethesda, ML, USA).

### 2.6. Western Blot Analysis

Pro-Prep Protein Extraction Solution (Intron Biotechnology Inc., Seongnam, Republic of Korea) was used according to the manufacturer’s protocol to prepare total proteins from the wound skin. Protein homogenates were centrifuged at 13,000 rpm at 4 °C for 5 min, after which the total protein concentrations were determined using a SMARTTM Bicinchoninic Acid Protein assay kit (Thermo Fisher Scientific Inc., Waltham, MA, USA). Approximately 20 µg of the proteins were subjected to 8–10% sodium dodecyl sulfate–polyacrylamide gel electrophoresis (SDS-PAGE) for 2 h, and the resolved proteins were transferred to nitrocellulose membranes for 3 h at 40 V. The proteins were then transferred to a nitrocellulose membrane (Amersham Biosciences, Corston, UK) for 2 h at 40 V in transfer buffer (25 × 10^−3^ M Trizma-base, 192 × 10^−3^ M glycine, and 20% methanol). The appropriate dilutions of the primary antibodies, anti-COL1A1-antibody (Cell signaling Inc., Danvers, MA, USA), and anti-PDGF-BB antibody (Thermo Fisher Scientific Inc., Waltham, MA, USA) were added to the protein-transferred membranes and allowed to hybridize overnight at 4 °C. After washing three times with a solution composed of 10 × 10^−3^ M Trizma base (pH 7.6), 150 × 10^−3^ M NaCl, and 0.05% Tween-20, the membrane was incubated with a horseradish peroxidase-conjugated secondary antibody for 1 h at room temperature. Membranes were developed using an enhanced chemiluminescence detection system (Amersham Bioscience, Corston, UK). Finally, the results were quantified using an Image Analyzer System (Eastman Kodak 2000MM, Rochester, NY, USA) and expressed as the fold increase over the values of the Control group based on β-actin expression as an internal control.

### 2.7. Quantitative Real-Time—Polymerase Chain Reaction (RT-qPCR) Analysis

The frozen skin tissue was briefly homogenized in RNA Bee solution (Tet-Test; Friendswood, TX, USA). Total RNA was isolated using centrifugation at 15,000 rpm for 15 min, after which the RNA concentration was measured using a NanoDrop Spectrophotometer (Allsheng, Hangzhou, China). Approximately 4 µg of the total RNA was annealed with 500 ng of oligo-dT primer (Thermo Fisher Scientific Inc., Waltham, MA, USA) at 70 °C for 10 min. Complementary DNA (cDNA) was synthesized using Superscript II Reverse Transcriptase (Invitrogen, Carlsbad, CA, USA). RT-qPCR was performed with the cDNA template obtained (2 µL) and 2 × Power SYBR Green (6 µL; Toyobo Life Science, Osaka, Japan) containing the following specific primers: IL-1β, sense primer 5′-GCACA TCAAC AAGAG CTTCA GGCAG-3′ and anti-sense primer: 5′-GCTGC TTGTG AGGTG CTGAT GTAC-3′; TNF-α, sense primer: 5′-CCTGT AGCCC ACGTC GTAGC-3′ and anti-sense primer: 5′-TTGAC CTCAG CGCTG ACTTG-3′; TGF-β1, sense primer 5′-GAGGT CACCC GCGTG CTA-3′ and anti-sense primer 5′-TGTGT GAGAT GTCTT TGGTT TTCTC-3′; IL-6, sense primer 5′-CTCTCTGCAAGAGAGTTCCATCCAG-3′ and anti-sense primer 5′- GCTATGGTACTCCAGAAGACCAGAGG-3′; β-actin, sense primers 5′-ACGGC CAGGT CATCA CTATT G-3′ and anti-sense primers 5′-CAAGA AGGAA GGCTG GAAAA GA-3′. The RT-qPCR was performed for 40 cycles using the following sequence: denaturation at 95 °C for 15 s, annealing at 57 °C for 15 s, and extension at 70 °C for 60 s. The fluorescence intensity was measured at the end of the extension phase of each cycle. The threshold values for the fluorescence intensities of all samples were set manually. The reaction cycle at which the PCR products exceeded this fluorescence intensity threshold during the exponential phase of PCR amplification was considered the threshold cycle (Ct). Expression of the target gene was quantified relative to the housekeeping gene β-actin, based on a comparison of the Cts at a constant fluorescence intensity according to Livak and Schmittgen’s method [24].

### 2.8. Statistical Significance Analysis

Statistical analyses were performed using SPSS release 10.10 for Windows (IBM SPSS, SPSS Inc., Armonk, NY, USA). The significance of intergroup differences was determined using one-way analysis of variance, followed by Tukey’s post-hoc test for multiple comparisons. Data are presented as means ± standard deviations, and statistical significance was accepted for *p* values < 0.05.

## 3. Results

### 3.1. Effect of Treated SiNP Concentration on Adhesion between PDMA Hydrogel

To investigate the effect of the SiNP treatment on the adhesion between the two substrates, a lap shear test was performed, as shown in Figure 1a. The SiNPs were treated on one side of the substrate and adhered to the other side of the substrate. The size and smooth spherical morphology of SiNP_10_ (9.6 ± 1.5 nm in diameter) and SiNP_30_ (29.3 ± 3.8 nm in diameter) were observed in Figure 1b and Figure 1c, respectively, characterized by SEM images. The degree of adhesion between the PDMA hydrogels with different concentrations of SiNPs was evaluated. Polyacrylamide hydrogel with the same cross-linking density and similar degree of swelling is unable to bear weight, even when subjected to significant pressure, and does not adhere to SiNPs. Moreover, when poly (ethylene oxide) hydrogels were adsorbed onto the SiNP surface, they exhibited low interaction and adsorption binding energy [25,26,27]. In contrast, the PDMA hydrogel exhibits ease of adsorption and can energetically interact with the silica surface.

As the concentration of the treated SiNPs increased, both the force and displacement increased and then diminished (Figure 1d–f). At above 20 wt% SiNP treatment, the texture between PDMA hydrogel became slippery because the high concentration of SiNP provided multilayers on the surface of the hydrogel, preventing efficient bridging between PDMA hydrogels. The maximum adhesion strength between the PDMA hydrogels after SiNP_10_ treatment was higher than that after SiNP_30_ treatment at all tested concentrations (Figure 1f). To compare the size effect of the SiNPs on adhesion in detail, the optimal concentration of the treated SiNPs was set to 10 wt% as the value of the saturated maximum adhesive strength for further studies.

### 3.2. Effect of Treated SiNP Size on Adhesion between Tissue Model

**Figure 2:**
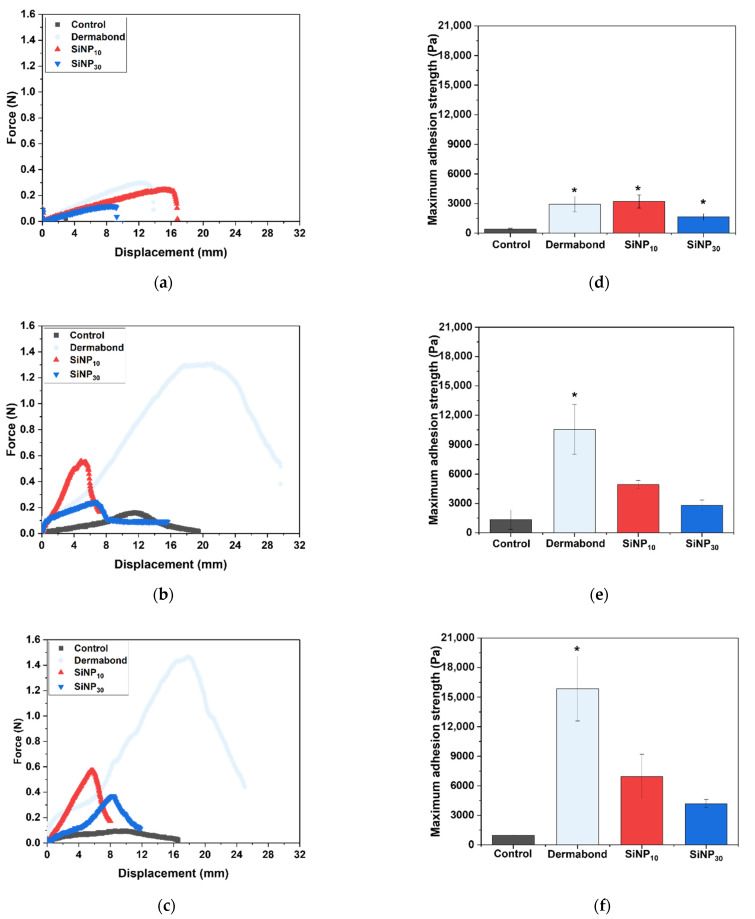
Lap shear adhesion tests using SiNP treatment between various substrates. Representative displacement-force curves obtained from the adhesion tests depending on the size of 10 wt% SiNP treatment between two substrates of (**a**) gelatin, (**b**) porcine liver, and (**c**) porcine heart. Maximum adhesion strength depending on the size of SiNP treated between two substrates of (**d**) gelatin, (**e**) porcine liver, and (**f**) porcine heart. The data (*n* = 3) were presented as mean ± SD. * *p* < 0.05 vs. Control group.

To observe the adhesive profile between various substrates beyond the PDMA hydrogel, the displacement force was measured using a skin tissue-like gelatin hydrogel, porcine liver, and porcine heart. The SiNP-treated groups (SiNP_10_ and SiNP_30_) showed higher maximum adhesion strengths than the Control group (not addressed between the substrates) (Figure 2). As a clinical control, the adhesion test using Dermabond was performed; the maximum adhesion strength was the highest among experimental groups, and the standard deviation values were correspondingly high. There was no significant difference between the adhesion ability of SiNP_10_ and SiNP_30_ treatments on the gelatin hydrogel; however, the maximum adhesion strength using SiNP_10_ (3206 ± 670 Pa) and SiNP_30_ (1633 ± 320 Pa) treatments was approximately 7.58 and 3.86 times higher, respectively, than that of Control group (423 ± 81 Pa) based on average value.

In the case of the porcine liver, the maximum adhesion strength obtained using the SiNP_10_ (4928 ± 429 Pa) and SiNP_30_ (2805 ± 544 Pa) treatment was approximately 3.71 and 2.11 times higher, respectively, than that of Control group (1330 ± 992 Pa) based on average value. In the porcine heart, the maximum adhesion strength of SiNP_10_ (6955 ± 2265 Pa) and SiNP_30_ (4183 ± 417 Pa) treatment was approximately 7.15 and 4.30 times higher, respectively, than that of Control group (973 ± 22 Pa) based on average value.

### 3.3. Effect of SiNP Treatment on Skin Incision Wound Closing

**Figure 3:**
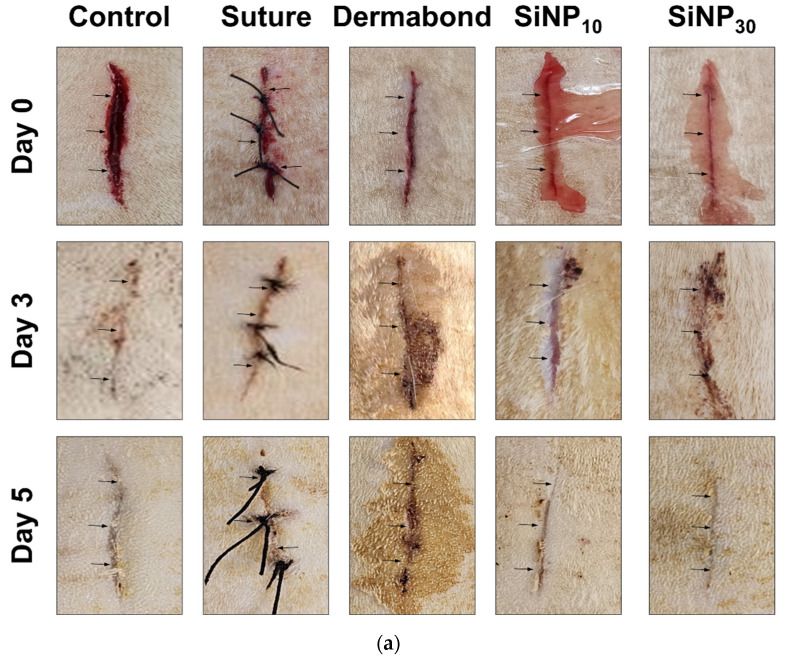

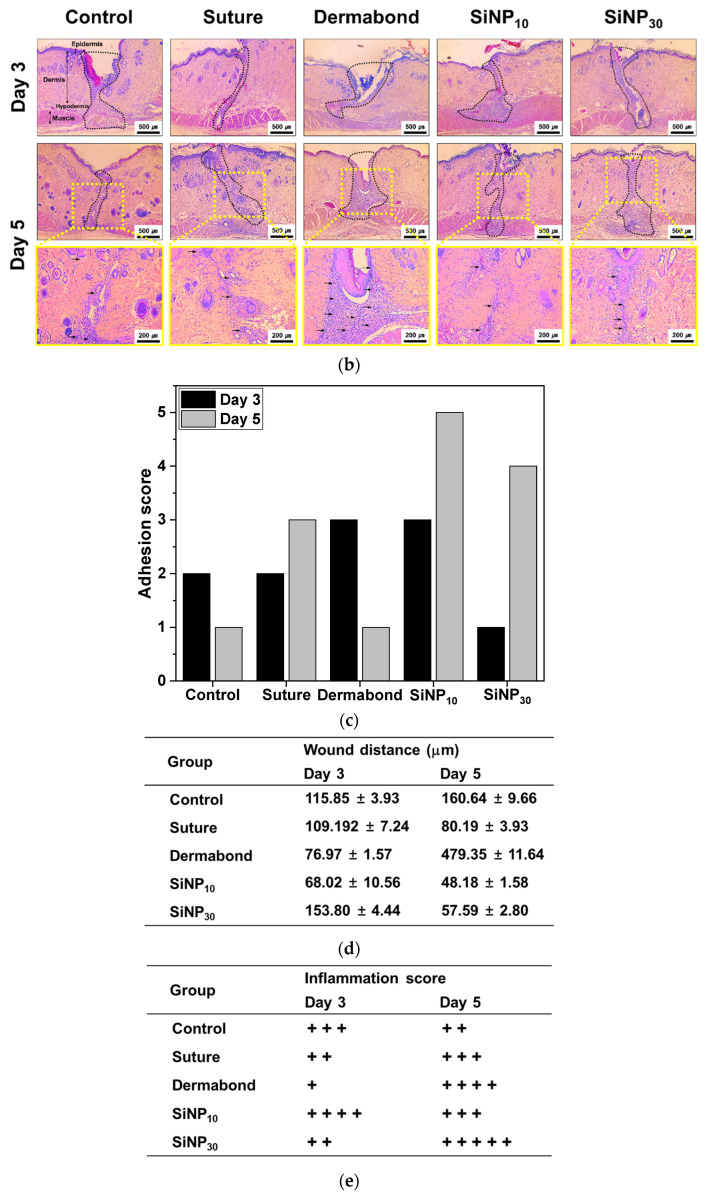
Morphology and histological images of incision wound skin after treatment of SiNP. (**a**) Images of the wound area of the subset group were captured by a digital camera on days 3 and 5. (**b**) Adhesion and inflammation were observed in H&E skin sections at 100× and 400× magnification. The adhesion regions in incision wound skin were presented with a black dotted line. Arrow indicated inflammation sites. The preparation of the H&E stained tissue sections was performed on five to six rats per group; the pathological factors were analyzed twice for each stained tissue. (**c**) Adhesion scores (from 1 to 5 level), (**d**) wound distance, and (**e**) inflammations scores in H&E-stained sections of wound tissue. Experimental groups are described as follows: Control: nothing addressed into the incision wound, Suture: sutured the incision wound with suture, Dermabond: glued together in regions of incision wound with commercial cyanoacrylate glue (DermabondTM mini), SiNP_10_ and SiNP_30_: attached with 10 nm and 30 nm in diameter-sized silica nanoparticles solution. Adhesion scoring; 1: nearly non-adherent, 2: some layers attached including empty space, 3: some layers attached, 4: most of the layers are attached, 5: all layers fully adherent. Inflammation scoring; +: presence of Very few inflammatory cells, + +: presence of few inflammatory cells, + + +: moderate inflammatory cells, + + + +: many inflammatory cells, + + + + +: exaggerated inflammatory cellularity.

To determine whether SiNPs affect wound closure and tissue adhesion in the incision wound skin, alterations in wound morphological features and histopathological structure were analyzed in the incision wound skin of SD rats after treatment with SiNPs for 3 and 5 days. The wound closure rate was significantly higher in the SiNP_10_ and SiNP_30_ groups than that in the other groups on day 5, although there was no significant difference on day 3 (Figure 3a). Similar effects were observed in the histopathological structure of the wounded skin on day 5 (Figure 3b). Between days 3 and 5, the adhesion score markedly increased in the SiNP groups (SiNP_10_: from + + + to + + + +; SiNP_30_: from + to + + + +) compared to the Control (from + + to +), Suture (from + + to + + +), and Dermabond groups (from + + + to +) (Figure 3c). Also, this score was successfully supplemented with wound distance (Figure 3d). The SiNP_10_ group showed a higher adhesion score than the Dermabond group, whereas the inflammation score on day 5 was lower in the SiNP_10_ group (+ + +) than in the SiNP_30_ group (+ + + + +) (Figure 3e). These results showed that SiNP_10_ promoted wound closure and tissue adhesion and reduced inflammation in the incision wound skin of SD rats.

### 3.4. Effect of SiNP Treatment on Inflammatory Response from Wounded Skin

**Figure 4:**
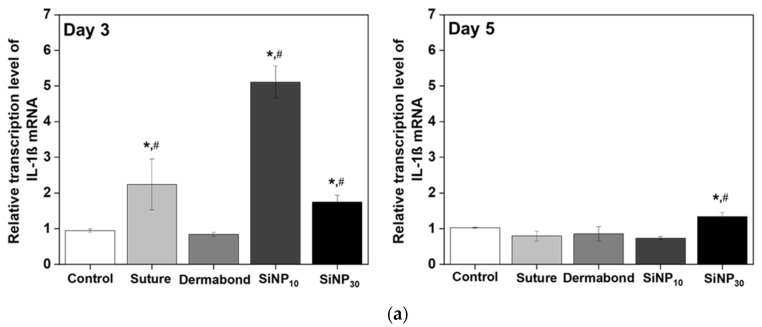

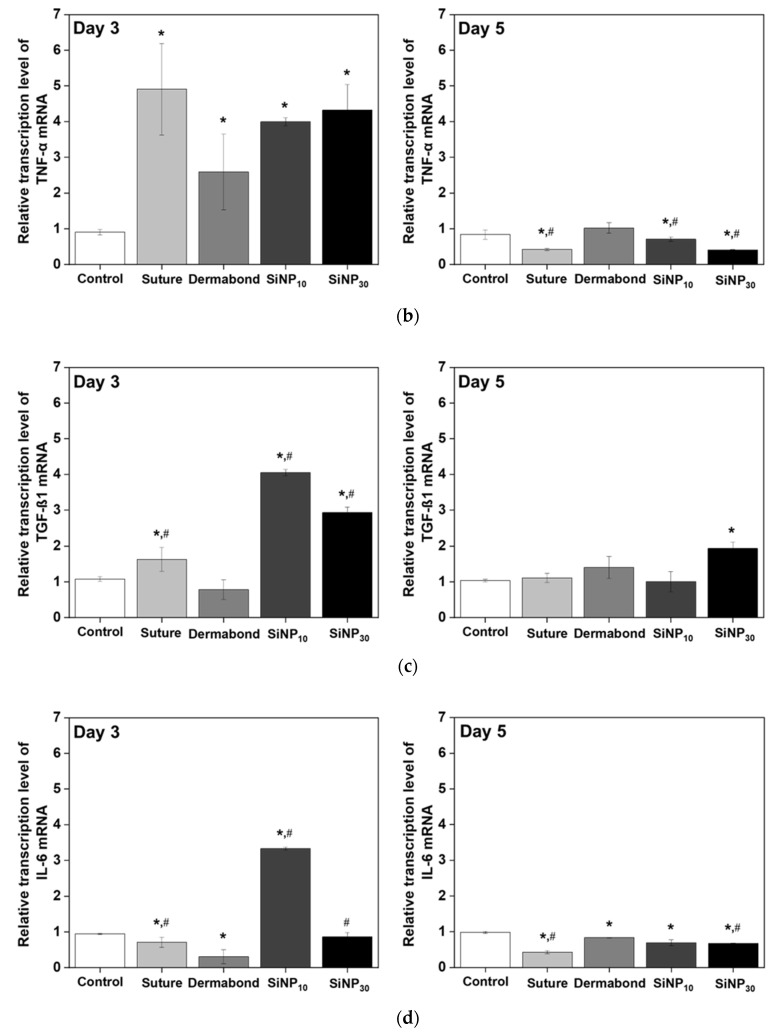
Transcription level of inflammatory cytokines in incision wound skin after treatment of SiNP. The levels of (**a**) IL-1β, (**b**) TNF-α, (**c**) TGF-β1, and (**d**) t6 transcripts in the total mRNA of mid-colons were measured by RT-qPCR using specific primers. The mRNA levels of three genes were calculated, based on the intensity of β-actin as an endogenous control. The total RNA analysis was performed on three to five mice per group and the RT-qPCR analyses were assayed twice for each total RNA. The data were presented as mean ± SD. * *p* < 0.05 vs. Control group. # *p* < 0.05 vs. Dermabond group. Abbreviations: IL, Interleukins; TNF, Tumor necrosis factors; TGF, Transforming growth factor.

To investigate whether the promoting effects of SiNPs on wound closure and tissue adhesion were accompanied by changes in the inflammatory response, alterations in the expression levels of inflammatory cytokines were measured in the incision wound skin of SD rats after treatment with SiNPs for 3 and 5 days. On day 3, the expression levels of four cytokines, including IL-1β, TNF-α, TGF-β1, and IL-6, increased in the SiNP_10_ and SiNP_30_ groups compared to the Control and Dermabond groups (Figure 4). However, at day 5, these increased levels at day 3 were significantly decreased in SiNP_10_ group (IL-1β: from 5.11 ± 0.45 to 0.74 ± 0.04; TNF-α: from 3.99 ± 0.11 to 0.71 ± 0.06; TGF-β1: from 4.05 ± 0.09 to 1.01 ± 0.28; IL-6: from 3.33 ± 0.04 to 0.7 ± 0.08); compared with Control (IL-1β: from 0.95 ± 0.04 to 1.02 ± 0.02; TNF-α: from 0.91 ± 0.08 to 0.84 ± 0.13; TGF-β1: from 1.08 ± 0.07 to 1.04 ± 0.03; IL-6: from 0.95 ± 0.02 to 0.98 ± 0.03) or Dermabond group (IL-1β: from 0.84 ± 0.0 to 0.86 ± 0.2; TNF-α: from 2.6 ± 1.06 to 1.02 ± 0.15; TGF-β1: from 0.79 ± 0.27 to 1.4 ± 0.31; IL-6: from 0.31 ± 0.2 to 0.83 ± 0.01). The SiNP_30_ group showed a significant decrease in the expression level of TNF-α at day 5 compared to that in the Control group.

### 3.5. Effect of SiNPs Treatment on Tissue Regeneration and Connective Tissue Formation from the Wounded Skin

**Figure 5:**
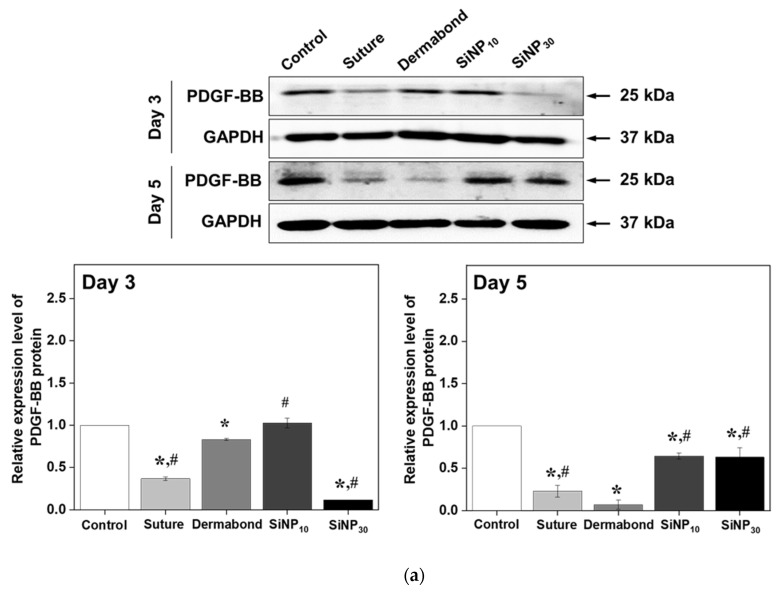

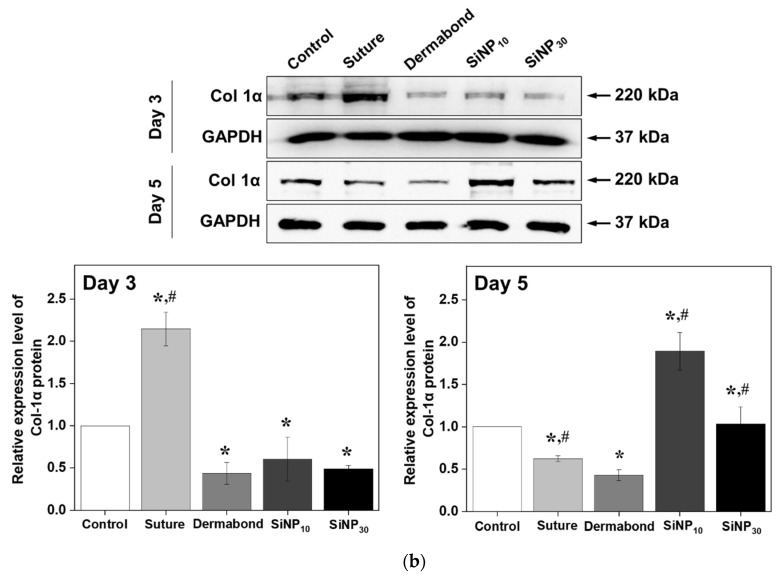
Expression level of key regulators for angiogenesis and the formation of connective tissue in incision wound skin after treatment of SiNP. (**a**) Expression level of PDGF-BB proteins. (**b**) Expression level of Col 1α proteins. After collecting the wound skin, the protein expression levels of the PDGF-BB proteins and Col 1α proteins were analyzed using specific antibodies and densitometry. The tissue homogenates were prepared from three to five mice per group and western blot was analyzed twice for each sample. The level of each protein was normalized to β-actin. The data were presented as mean ± SD. * *p* < 0.05 vs. Control group. # *p* < 0.05 vs. Dermabond group. Abbreviations: PDGF-BB, Platelet-derived growth factor-BB; Col, Collagen.

To investigate whether the promoting effects of SiNP on wound closure and tissue adhesion were accompanied by changes in angiogenesis and the formation of connective tissue in the wound skin, alterations in the expression levels of PDGF-BB and Col-1a were measured in the incision wound skin of SD rats after treatment with SiNP for 3 and 5 days (Figure 5). The expression levels of PDGF-BB proteins at day 5 were significantly higher in SiNP_10_ (0.64 ± 0.03) and SiNP_30_ group (0.63 ± 0.11) compared to the Dermabond group (0.07 ± 0.05) although this level at day 3 was similarly maintained between Dermabond (0.83 ± 0.01) and SiNP_10_ (1.03 ± 0.06) (Figure 5a). A similar pattern was detected for the expression levels of Col-1a protein on day 5. The expression level of Col-1a proteins at day 5 was higher in SiNP_10_ (1.9 ± 0.22) and SiNP_30_ group (1.03 ± 0.2) than Dermabond group (0.43 ± 0.06) although their level was constant remained in three groups at day 3 (SiNP_10_: 0.61 ± 0.26, SiNP_30_: 0.5 ± 0.04, Dermabond: 0.44 ± 0.13) (Figure 5b). Therefore, these results, which would contribute to stimulating angiogenesis and the formation of connective tissue in the incision wound skin of SD rats, may be associated with the promoting effects of SiNP_10_ on wound closure and tissue adhesion compared to the Dermabond group.

## 4. Discussion

The interfacial area of treated nanoparticles is a critical factor in determining the adhesive strength with the polymer of hydrogels [17]. At the same concentration, SiNP_10_ exhibits a higher total external surface area compared to SiNP_30_ and has a diameter similar to the network mesh size of PDMA hydrogels, enabling easy adhesion without chemical reactions [17,20]. Notably, the high surface area of SiNP_10_ allows for multiple strands to adsorb rather than a single strand [28], ensuring strong attachment and acting as a bridge between particles [20], thus preventing easy detachment [29]. In the case of SiNP_10_, the particle size was smaller than that of SiNP_30_, and the surface area available for adsorption with the polymer chain of the PDMA hydrogel was larger [18]. However, for SiNP_30_, saturation was reached earlier as the concentration increased, indicating that fewer polymer chains were available to bond the inorganic particles. Therefore, the interaction between the polymer chain of the hydrogel and SiNPs was reduced, and the adhesive strength did not significantly increase [25]. In summary, more polymer chains are adsorbed onto the surface of SiNP_10_, resulting in enhanced adhesion.

Similar effects on the wound morphological features and histopathological structure of the incision wound skin were detected in previous studies using other types of SiNPs, although there were differences in the animals and analytical factors used. Treatment with sub-100 nm colloidal mesoporous silica (CMS) particles did not induce any significant leakage, infection, or inflammatory reactions on the wounded skin of BalB/c mice on days 3 and 5, but showed a higher arrangement of epidermal layers compared to the control group [18]. In addition, complete sealing, recovery of epidermal thickness, and a significant reduction in scar width were observed in the wound skin of SD rats within 8 days of treatment with ceria nanocrystal-decorated mesoporous silica nanoparticles (MSN-Ceria) [30]. Furthermore, the spread of the synthesized SiNPs into the full-thickness dorsal skin injury of Wistar rats induced complete wound closure without pathological inflammation or necrosis [20].

Therefore, the above results suggest that the promoting effects of SiNP_10_ on wound closure and tissue adhesion may be linked to the suppression of inflammation during the wound healing process. In detail, compared with Suture or Dermabond group, the lower viscosity SiNP solution penetrated entirely into incision wounds, and was located on the internal tissues of the wounds. That meant that the SiNP could stimulate the accumulation of neutrophils and macrophages as well as the release of inflammatory and growth factors at the inflammation stage (within day 3) of wound healing [31,32]. Accordingly, the SiNP treatment promoted the transcription of inflammatory cytokines on day 3 after injury. Moreover, we observed the dramatic reduction of inflammatory cytokines at the proliferation state including angiogenesis, proliferation, and epithelization of the wound healing process after a day. The similar trend of changes in inflammatory cytokines during the wound healing process could be observed in previous studies [33,34,35]. Silver nanoparticles treatment raised inflammatory cytokines, e.g., TGF-β1 was higher in the initial period (up to 3 days) of healing than that of the non-treated group. Then, the expression of TGF-β1 was decreased and maintained at a lower level during the latter phase of healing (from day 5 to day 30).

However, no direct analysis of inflammatory cytokines has been performed on SiNP-treated skin wounds. To the best of our knowledge, only a few studies have reported the inflammatory response in SiNP-treated wounded skin based on the morphological characteristics of wound appearance [18,20]. Therefore, the present study provides the first evidence of the suppressive effects of inflammatory cytokines during the latter phase of wound healing on incision wound skin by SiNP treatment.

Previous findings on angiogenesis and the formation of connective tissue in SiNP-treated wounded skin were very similar to our findings. The density of collagen on Masson’s trichrome-stained wound skin significantly increased after treatment with CMS particles [18]. Additionally, the level expression of platelet-derived growth factor-a (PDGF-a), an essential growth factor for cell proliferation, migration, and division [36], increased markedly in the MSN-Ceria treated group compared to the control group [30]. In the present study, we provided quantitative results for the first time by directly measuring the amounts of PDGF and Col proteins to overcome the limitations of previous studies.

The SiNP treatment affects the metabolic activity of cells regardless of the dosage and incubation time, and, in a scratch assay, silica treatment promoted wound closure. The SiNPs facilitate wound closure by providing a nano-bridging technology that allows the sealing and healing of deep wounds without complex procedures [11,20,37,38,39]. In addition, they assist in the proliferation of skin fibroblasts and their migration to the injured area, releasing silicic acid and improving in vitro localized wound healing [40,41].

## 5. Conclusions

The effect of SiNP treatment on the adhesion between various tissue-like substrates was observed to depend on the size and concentration of the SiNPs. The enhanced contribution of SiNP treatment to in vivo wound closure and healing in rat skin was demonstrated. The SiNP treatment not only exhibited stronger wound closure but also inflammatory cytokines during the latter phase of wound healing on incision wound sites compared to the suturing and commercial skin adhesive-treated groups. These effects are closely associated with the promotion of angiogenesis and connective tissue formation, which contribute to enhanced wound healing. The potential efficacy of tissue regeneration suggests its application for extending chronic wound care beyond acute wounds.

## Data Availability

The original contributions presented in the study are included in the article, further inquiries can be directed to the corresponding authors.
